# Robust Classification Using Posterior Probability Threshold Computation Followed by Voronoi Cell Based Class Assignment Circumventing Pitfalls of Bayesian Analysis of Biomedical Data

**DOI:** 10.3390/ijms232214081

**Published:** 2022-11-15

**Authors:** Alfred Ultsch, Jörn Lötsch

**Affiliations:** 1 DataBionics Research Group, University of Marburg, Hans-Meerwein-Straße 22, 35032 Marburg, Germany; 2 Institute of Clinical Pharmacology, Goethe-University, Theodor-Stern-Kai 7, 60590 Frankfurt am Main, Germany; 3 Fraunhofer Institute for Translational Medicine and Pharmacology ITMP, Theodor-Stern-Kai 7, 60596 Frankfurt am Main, Germany

**Keywords:** data science, artificial intelligence, machine learning, digital medicine

## Abstract

Bayesian inference is ubiquitous in science and widely used in biomedical research such as cell sorting or “omics” approaches, as well as in machine learning (ML), artificial neural networks, and “big data” applications. However, the calculation is not robust in regions of low evidence. In cases where one group has a lower mean but a higher variance than another group, new cases with larger values are implausibly assigned to the group with typically smaller values. An approach for a robust extension of Bayesian inference is proposed that proceeds in two main steps starting from the Bayesian posterior probabilities. First, cases with low evidence are labeled as “uncertain” class membership. The boundary for low probabilities of class assignment (threshold ε) is calculated using a computed ABC analysis as a data-based technique for item categorization. This leaves a number of cases with uncertain classification (*p* < ε). Second, cases with uncertain class membership are relabeled based on the distance to neighboring classified cases based on Voronoi cells. The approach is demonstrated on biomedical data typically analyzed with Bayesian statistics, such as flow cytometric data sets or biomarkers used in medical diagnostics, where it increased the class assignment accuracy by 1–10% depending on the data set. The proposed extension of the Bayesian inference of class membership can be used to obtain robust and plausible class assignments even for data at the extremes of the distribution and/or for which evidence is weak.

## 1. Introduction

**Figure 1:**
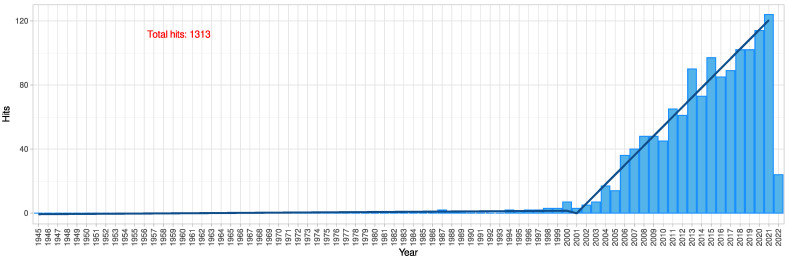
Bar chart of the number of biomedical publications per year obtained by a computational search of the PubMed database for “(naive Bayesian OR naive Bayesian classifier OR probabilistic classifiers) NOT review [Publication Type]”. The line shows the bilinear trend in the number of publications with an acceleration from year 2001. The figure has been created using the software package R (version 4.1.3 for Linux; https://CRAN.R-project.org/ (accessed on 18 September 2022) [12] accessed on 18 September 2022 and the R library “ggplot2” (https://CRAN.R-project.org/package=ggplot2 (accessed on 18 September 2022) [13]).

Statistical Bayesian reasoning has been successfully applied to a wide range of applications. It has been used for 250 years [1]. It is a standard used in workflows and implemented in software environments for the analysis of biomedical and other data. Cell sorting and “omics” research are among the molecular biomedical applications where Bayesian inference is used as a default component of many workflows [2,3,4,5]. Modeling data distributions with mixture models is also commonly used in machine learning. The task of assigning cluster labels to the data, i.e., classification, is usually performed using Bayes’ theorem. Bayesian inference is used almost ubiquitously in science and other areas of human activity. For its use in biomedical research, a PubMed database search at https://pubmed.ncbi.nlm.nih.gov/ accessed on 29 March 2022, using the R library “RISmed” (https://CRAN.R-project.org/package=RISmed (accessed on 18 September 2022) [6]) and the search string “(naive Bayesian OR naive Bayesian classifier OR probabilistic classifiers) NOT review [Publication Type]” returned 1313 hits with a pronounced increase in yearly publications during the last two decades (Figure 1). Drawing inferences using Bayes’ theorem has recently been increasingly used in machine learning to realize artificial intelligence algorithms [7]).

**Figure 2:**
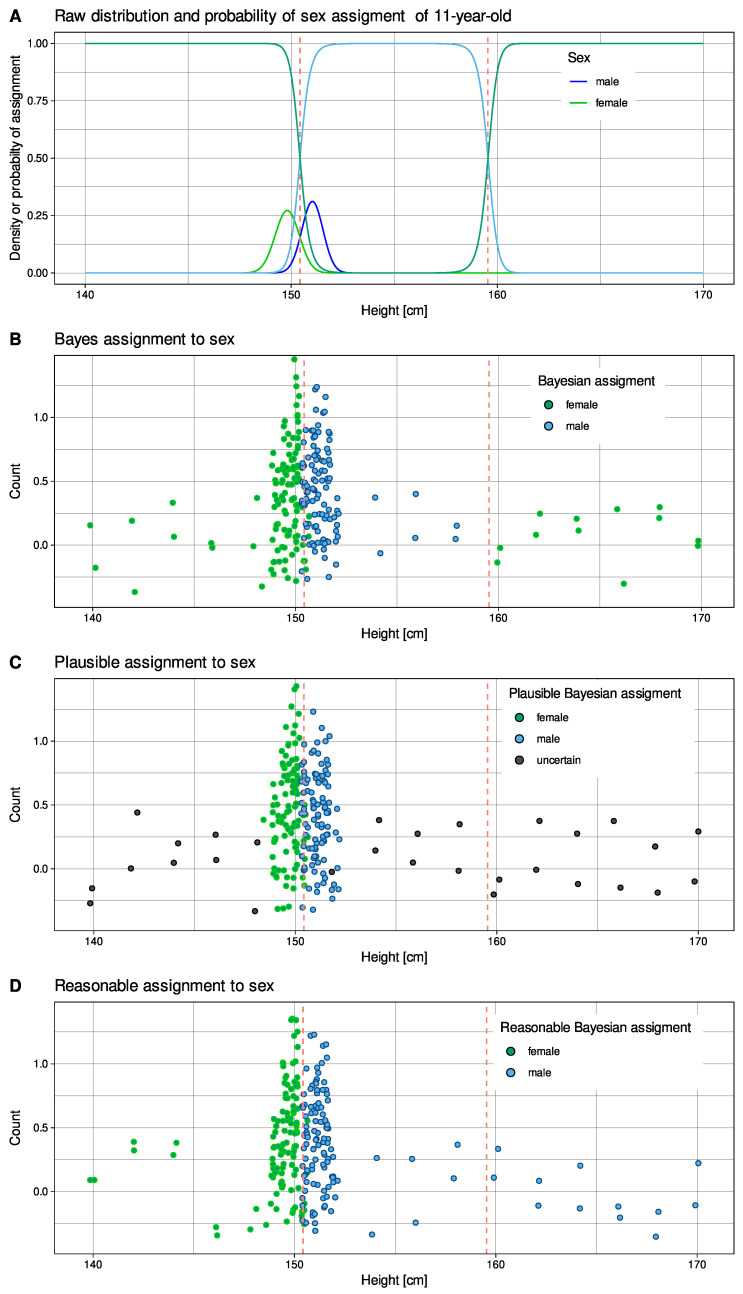
Distribution of body heights 11-year-old girls (red) and boys (blue) and assignment to sex based on Bayesian posteriors of observations of sex-specific body height [9]. (**A**) Probability density functions based on observed mean and standard deviation values of 149.8081 ± 0.5843 cm for girls and 151.0295 ± 0.5108 cm for boys. In addition, Bayesian posterior probabilities of sex assignment are shown in lighter blue and red lines. Bayesian decision boundaries are shown as vertical red dashed lines. (**B**) Simulation of 100 heights per sex randomly drawn from a normal distribution with above means and standard deviations, and addition of 16 arbitrary heights between 140 and 170 cm in increments of 2 cm. Points are colored for assigned sex based on Bayesian posteriors shown in panel A. The points are jittered for better distinguishability. (**C**) Following the method of reasonable Bayes proposed in this report, cases with certain versus uncertain class assignment were identified, which occurred in marginal regions where the probability density functions of the data classes were very low. (**D**) For cases unclassified by the above method, sex assignment was substituted based on the “plausible Bayes” method proposed in this report. The figure has been created using the R software package (version 4.1.3 for Linux; https://CRAN.R-project.org/ (accessed on 18 September 2022) [12] (accessed on 18 September 2022) and the R library “ggplot2” (https://CRAN.R-project.org/package=ggplot2 (accessed on 18 September 2022) [13]).

However, reasoning with Bayes’ theorem is not robust in regions of low evidence [8]. One of the undesirable effects is the possibility of implausible class labeling. Consider, as an example, data on body size. In a recent summary of height and body mass index of school-aged children and adolescents between 1985 and 2019 with a total of 65 million participants [9], data for 11-year-old Germans showed that girls were normally distributed with a mean ± standard deviation of 149.8081 ± 0.5843 cm. Boys were also Gaussian distributed with a height of 151.0295 ± 0.5108 cm. Applying Bayes’ theorem to values of height from 140 to 170 cm correctly assigned the female class for values less than 151 cm and the male class for the range 152 to 159 (Figure 2A). However, individuals with heights greater than 160 cm were classified by Bayes statistics as definitive female (Figure 2B). The reason for this effect is that the variance of the distribution of girls was larger than that of boys. This led to larger values of the probability distribution (pdf) of girls compared to boys over 160 cm. Bayesian reasoning thus became implausible at large heights because it concluded that “all giants are female”. Depending on the structure of covariances, Bayesian statistics, i.e., the strong decision to assign a class based on a high posterior probability occasionally collides with the low probability of cases, i.e., low evidence. Moreover, a minimum change in variance can cause a maximum change in the probability of class assignment that reverses the decision, and variances can be difficult and error-prone to estimate from the data [10]. This calls into question the robustness of Bayes’ theorem including robust approaches based on it [11].

## 2. Results

### 2.1. Multiple Sclerosis Lipidomics Data

**Figure 3:**
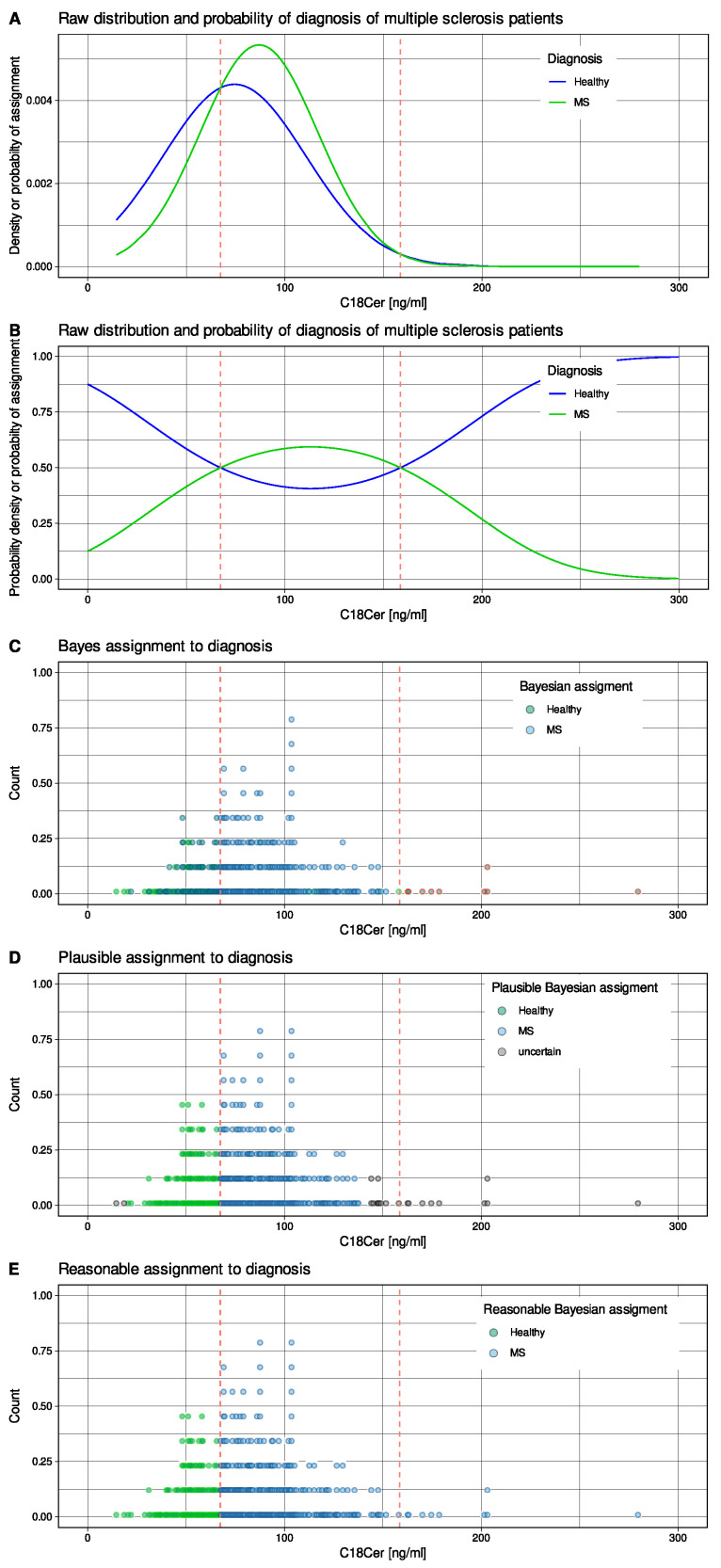
Distribution of serum concentrations of the lipid mediator C18Cer of the ceramide class, assayed in the serum of n = 102 patients with multiple sclerosis and n = 301 healthy controls [14]. (**A**) Probability density functions based on observed means and standard deviations of 74.3881 ± 36.2643 ng/mL for patients with multiple sclerosis and 86.8806 ± 29.8202 for healthy subjects. (**B**) Bayesian posterior probabilities for assignment to patients or controls are shown in light blue and red lines. Bayesian decision boundaries are shown as vertical red dashed lines. (**C**) Dot histogram representation of individual cases. Dots are colored for multiple sclerosis or healthy diagnosis based on Bayesian posteriors in panel B. (**D**) According to the “reasonable Bayes” method proposed in this report, cases with uncertain class assignment were identified, which occurred in marginal areas where the probability density functions of the data set instances were very low. (**E**) For cases that were not classified by the above method, assignment to clinical diagnosis group was based on the “plausible Bayes” method proposed in this report. The figure has been created using the R software package (version 4.1.3 for Linux; https://CRAN.R-project.org/ (accessed on 18 September 2022) [12]) and the R library “ ggplot2” (https://cran.r-project.org/package=ggplot2 (accessed on 18 September 2022) [13]).

Serum concentrations of n = 43 different lipid markers of different classes (ceramides, lysophosphatidic acids, endocannabinoids, pterins, prostaglandins, dihydroxyeicosatrienoic acids, and HETEs) were available from n = 102 patients with multiple sclerosis and from n = 301 healthy subjects previously published [14]. Concentrations were analyzed by liquid chromatography–electrospray ionization tandem mass spectrometry (LC-ESI-MS/MS) [15,16]. One of the ceramides, C18Cer, was selected for the present calculations. C18Cer plays a critical role in fat-induced insulin resistance in skeletal muscle [17] and has been implicated in multiple sclerosis where it was statistically significantly correlated with the Expanded Disability Status Scale [18]. It was not included in a sensitive, complex, supervised, machine-learning-based lipid biomarker for multiple sclerosis; however, it showed the same pathology of means and standard deviations between the two groups as the body weight data in the introductory example, namely that very low and very high values were assigned to the same group when Bayesian statistics were applied (Figure 3).

Patients with multiple sclerosis had, on average, lower C18Cer levels than healthy subjects (Figure 3A–E). The standard deviation in the empirical patient group happened to be higher than in the control group. The use of a Bayesian classifier had the undesirable effect of classifying individuals with C18Cer levels above 159 ng/mL as diseased, which would assign the diagnosis to subjects with particularly normal signals if C18Cer were a biomarker for multiple sclerosis. In the specific case of this experiment, this led to at least 1% of incorrect classifications. This was corrected when the classification was performed according to the proposed “plausible Bayes” strategy (Figure 3E). In a 100-fold repeated bootstrap experiment, the overall improvement of class assignment accuracy was small and raised from 68.8 ± 2% to 68.9 ± 2%; however, the nonparametric 95%-confidence interval moved from 64.9–74.1% for the Bayes classification to 64.8–73.8% for the class assignment according to the “reasonable Bayes” procedure.

### 2.2. Flow Cytometric Data

Flow cytometry with fluorescence-activated cell sorting (FACS) data were available from a hematologic analysis of n = 296,755 cells obtained from peripheral blood samples from n = 14 subjects (https://data.mendeley.com/datasets/jk4dt6wprv/1, accessed on 18 September 2022 [19]). FACS analyses originally included d = 10 features.

For the present experiments, d = 2 variables were used, including the differentiation cluster CD45, denoting protein tyrosine phosphatase, receptor type C, involved in B- and T-cell-receptor-mediated activation [20], and CD13, denoting membrane alanyl aminopeptidase N. CD13 is involved in the metabolism of regulatory peptides in various cell types, including, for example, macrophages and granulocytes, as well as involved in induced T cell activation [21], the response of leukemic cells to colony-stimulating factors [22] or the development of dendritic cells and macrophages from blood cells [23].

A FACS data analysis allows the easy identification of subgroups (classes, populations) of cells defined as Gaussian distributions in two-dimensional scatter plots [24]. Populations in the CD45/CD13 data set followed were identified as myeloid cells ( CD13+ CD45+), lymphocytes (CD13− CD45+) and nonleukocytes (CD13− CD45−). The assignment of cells to populations was done by autogating [25] supervised by a medical expert. Bayesian reasoning was performed using a Gaussian mixture model (GMM) with the expectation maximization (EM) algorithm [26]. EM selected three modes and optimized the parameters of the two-dimensional Gaussian distributions. The obtained GMM allowed the application of Bayes’ theorem to classify the data into the three classes defined by the likelihoods (pdf) for each event (cell). The three subpopulations could be defined as arbitrarily numbered classes, with class 1 characterized by low levels of expression of both CD45 and CD13, i.e., class 1 = CS45−, CD13−, class 2 = CD45+, CD13+, and class 3 = CD45+, CD13−.

**Figure 4:**
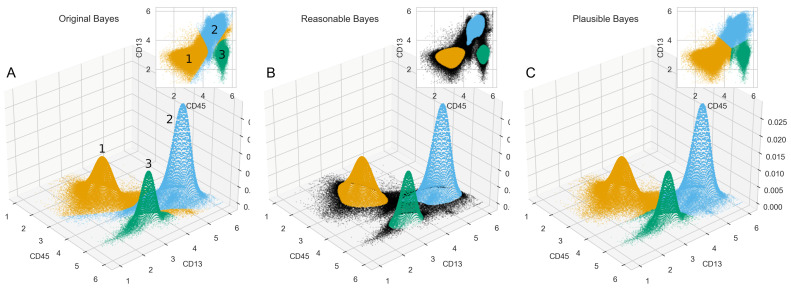
Three-dimensional dot plots of n = 296,755 cells from peripheral human blood analyzed by fluorescence-activated cell sorting (FACS) [19] and class assignment based on the Bayesian statistical method and modifications proposed in this report. Cells were labeled for d = 2 surface markers belonging to the CD45 and CD13 discrimination clusters. The distribution is modeled by three two-dimensional Gaussian curves whose parameters were fitted to the empirical data using the classical EM algorithm. Colors and class numbers are arbitrary for each class and indicate the assignment of each cell to one of the three modes based on (**A**) statistical reasoning using Bayes’ theorem or (**B**) “reasonable Bayes” classification as proposed here. Events shown in black have not been given a class label. (**C**) Plausible Bayes classification of the events, as suggested here: the Voronoi cells induced by the modes (= mean values) of the three Gaussian curves determine the class of the unlabeled data. For a precise localization of the classes, the insets show a top view of the data. The figure was created using Python version 3.8.12 for Linux (https://www.python.org (accessed on 18 September 2022)) and the “seaborn” statistical data visualization package (https://seaborn.pydata.org (accessed on 18 September 2022) [27]).

However, Bayesian statistical classification assigned cells with intermediate CD45 and CD13− to the CD13+ of class 2. Cells with high CD45 content (CD45+) that should be either class 2 or class 3 were assigned to class 1, resulting in 6% of the data apparently being incorrectly assigned to class 1 and appearing to be separated from the majority of the members of this class in the scatter plots (Figure 4A). Class 1, characterized by low CD45 levels (CD45−), had greater variance than the other two classes, so cells with CD45+ that fell between classes 2 and 3 were incorrectly assigned to class CD45−. This was circumvented by applying the “reasonable Bayes” calculation proposed here, in which no class assignment was made below a data-adjusted threshold (Figure 4B). The three Gaussian modes or the mean values of the Gaussian modes defined a Voronoi cell mosaic of level CC45 versus CD13. On this basis, the “plausible Bayes” calculation proposed here assigned the data that remained unclassified in the “reasonable Bayes” classification to the nearest class center (Figure 4C). In a 100-fold repeated bootstrap experiment, for cells with low empirical probabilities (<10 percentile of probabilities), the correct classes were assigned by Bayesian classification with an accuracy of 81.5 ± 0.3% and by “reasonable Bayes” classification with an accuracy of 91.4 ± 0.2%.

## 3. Discussion

Algorithms for classification, i.e., classifiers based on Bayes’ theorem, have become extremely popular in recent years in both statistics and machine learning. Their mention in the biomedical scientific literature has recently seen a strong upward trend. Using the Akaike information criterion [28] for a bilinear fit with a sliding breakpoint between segments, the trend in biomedical publications mentioning Bayesian methods can be described as accelerating from 2001, whereas prior to that year the number of publications was low (Figure 1).

The main reasons for this are, first, the ease of use of ready-made and predefined programs in most data analysis repositories or toolboxes such as CRAN (see above) or Bioconductor (https://bioconductor.org (accessed on 18 September 2022)) for R, the “Bioinformatics” Toolbox for Matlab, the “GaussianNB” method from “scikit-learn” (https://scikit-learn.org/stable/ (accessed on 18 September 2022) [29]) for Python, and many others. Second, there is theoretical evidence that Bayesian classifiers are optimal classifiers that minimize the probability of misclassification (e.g., [30]). Third, Bayesian classifiers (probabilistic classifiers) can be easily generalized to d dimensions, i.e., data sets with d variables (features, markers, parameters, measurements, dimensions). With the additional assumption of independence between features, the computation of Bayesian statistics is fast and simple, often linear in d, and still yields a high degree of accuracy [31,32].

The performance of Bayes classifiers, including those with independence assumption, also referred to as naïve or simple Bayes classifiers, has been reported to perform extremely well in many complex real-world situations, e.g., in classifying gene sequences [33], identifying lesions in the brain [34], including automatically identifying stroke lesions in MRI scans [35], and predicting the carcinogenicity of substances [36]. The Bayes classifier is considered one of the most popular classifiers for class prediction or pattern recognition for microarray gene expression data [37]. A theoretical analysis of classifiers based on Bayes’ theorem has shown that there are even good theoretical reasons for the apparently implausible effectiveness of probabilistic Bayes classifiers, even the naïve ones [36].

The method presented here considered regions of the data space where little was known about the data. One of the “curses of dimensionality” is that for multivariate data, almost all of the multidimensional space is empty [38]. Consider, for example, data on height and gender. Bayes’ theorem calculates with high certainty that giants, i.e., tall individuals, are female. This is related to the assumption of word proximity. If data (or assumptions) about giants were given, the theorem of Bayes would argue differently. Within the “closed world”, our approach either returns “don’t know” (“reasonable Bayes”) or uses the closest class (“plausible Bayes”). Since the mean body length of men (boys) is larger than that of women (girls), the “plausible Bayes” reasoning concludes that tall individuals are more likely to be male.

Implausible class assignments using Bayesian statistics can be detected in one or two dimensions by visualizing the data. For more than two features or dimensions, the most used Bayesian classifier is independent (naïve) Bayes using Gaussian mixture models (GMM). These models are also referred to as hidden Markov models (HMM) [39], Markov chains [40], or Kalman filters [41]. It can be assumed that many of the applications in multivariate statistics that use Bayesian inference suffer from the problem addressed in this report. The “plausible Bayes” approach proposed here avoids misclassification, while the “reasonable Bayes” calculation should be applied when distance calculations on high-dimensional empirical data are possible, and all data set instances need to be labeled.

## 4. Materials and Methods

### 4.1. Bayesian Reasoning

The theorem is well known, so its recapitulation can be minimized as follows. The starting point is the distribution of the observed data. Since the observed data in many cases result from sums of underlying processes, the assumption that the data follow a Gaussian distribution N (m,s) with parameters mean m and standard deviation s is justified by the central limit theorem [42]. This assumes that the data are generated by a process that uses this “likelihood function”. Returning to the introductory example of 11-year-old Germans [9], this means that 11-year-old boys in Germany are “produced” by nature using the sum of all parts of their bodies with an average height resulting in a Gaussian distribution N (m,s) with observed parameters m = 151.0295 cm and s = 0.5108 cm.

The second component of Bayes’ theorem is the probability of occurrence of a particular class of data, the so-called prior probability or “weight” of the class. In the present example the probability that a child is male. Under the so-called “closed world assumption” [43], i.e., there are only males and females, the weighted sum of the probabilities for boys and girls gives the evidence. This is the general probability to observe a data value. Thus, the probability of a child having a male sex as a function of its height can be calculated using Bayes’ theorem as $p\left(male|height\right)=p\left(male\right)\cdot \frac{likelihood\left(male\right)}{evidence}\;$.

Here, the likelihood is the “likelihood function” mentioned above, which follows the Gaussian distribution of body heights. The general form of this is $p\left(class|x\right)=p\left(class\right)\cdot \frac{likelihood\left(class\right)}{evidence}$. This formula for the Bayesian reasoning is critical for evidence close to zero. At such regions in the data space, the knowledge about the data’s classification is low (insecure), however, the posterior probabilities $p\left(class|x\right)$ suggest either 0 or 100% membership to one of the classes.

The posterior probability, i.e., the probability of having a certain sex, if the person has a certain height, is shown in Figure 2A (lines in the range 0 to 1). Below 150 cm, the probability of being a boy is low and approaches zero (see the left vertical red dashed line in Figure 2A). For individuals taller than 152 cm, the probability of being a boy is high and approaches *p* = 1 (see the right vertical red dashed lines in Figure 2). Bayes’ theorem can also be used to calculate a decision boundary, that is, a boundary that allows one to decide for or against a class, which in this example is at heights of 150.43 cm and 159.55 cm (red dashed lines in Figure 2). For heights of 11-year-old Germans below 159 cm, Bayes’ theorem holds perfectly and provides unambiguous assignments to the probable sex of the individual. However, a problem arises when the observation range is extended beyond 160 cm (Figure 2A, right side). When arguing with Bayes’ decision rule, one concludes that larger individuals must be female. This is a systematic error in Bayesian reasoning. It occurs when the lower (female) probability has a larger variance than the higher (male) probability, resulting in the probability being larger for a female than for a male beyond 160 cm. When visually inspecting the Bayesian reasoning as in Figure 2 or using interactive, visually guided software for Gaussian mixture analysis such as the R library “AdaptGauss” (https://CRAN.R-project.org/package=AdaptGauss (accessed on 18 September 2022) [44]), this can be detected. As will be shown below, the misclassifications can also be seen in appropriate visualizations in two dimensions. However, the implausibility of Bayesian results is difficult to detect for data with dimensions > 2. The results of such data mining, machine learning, and knowledge discovery applications, especially for biomedical data, may contain these types of obvious errors unnoticed.

In this report, we propose an approach to circumvent the implausible class assignment of observations by Bayesian statistics at the tails of the distribution of observations, where the probability of occurrence of a case is often very low and yet yields high posterior probabilities (Figure 2C). The proposed robust method first identifies the regions where class assignment is uncertain (“uncertain Bayes”) and then replaces the decision based on Bayesian statistics with a more plausible class assignment based on Voronoi cells [45] (Figure 2D).

### 4.2. Algorithm

**Figure 5:**
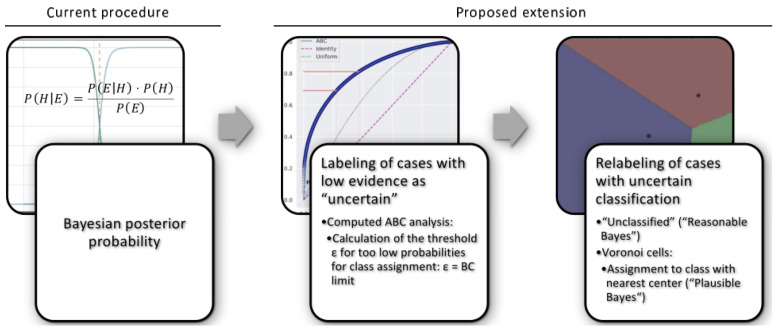
Proposed algorithm to circumvent misclassifications that occasionally occur when using the posterior probabilities in Bayes’ theorem that are very low. The threshold ε below which the evidence is considered as low is estimated for each pdf of the posteriors using a computed ABC analysis. Subset “C” of this item categorization contains the nonprofitable values, i.e., the lowest probabilities (“the trivial many”). These cases are classified as “uncertain”. Subsequently, a relabeling of these cases is performed, either as “unclassified”, which is referred to as “reasonable Bayes”, or they are assigned to the class with nearest class center based on Voronoi cell discrimination, which is referred to as “plausible Bayes”. Please note that the first row of panels shows schematic drawings similar to the main calculations in each step. The details are not explained. The second row of panels describes the individual steps. The figure was created using Microsoft PowerPoint^®^ (Redmond, WA, USA) on Microsoft Windows 11 running in a virtual machine powered by VirtualBox 6.1.36 (Oracle Corporation, Austin, TX, USA) as guest on Linux, and then further modified with the free vector graphics editor Inkscape (version 1.2 for Linux, https://inkscape.org/ (accessed on 18 September 2022)).

The proposed algorithm proceeds in two main steps starting from the Bayesian posterior probabilities (Figure 5). **First**, cases with low evidence are labeled as “uncertain” class membership. The boundary for low probabilities of class assignment (threshold ε) is calculated using a data-based technique for computational item categorization. This leaves a number of cases with uncertain classification (*p* < ε). **Second**, cases with uncertain class membership are relabeled based on the distance to neighboring classified cases.

#### 4.2.1. Calculation of the Threshold for Low Probabilities

Misclassifications using Bayes statistics occur when the posterior probabilities in Bayes’ theorem are very low, i.e., below a certain threshold ε. Using the computed ABC analysis, this limit ε can be estimated for each pdf for which n values pdf(x${}_{i}$) can be computed at equidistant supporting x${}_{i}$ with *i* = 1, …, n points within the data range of interest [46]. This method allows the optimal calculation of three disjoint subsets A, B, and C in data sets with positive values. Subset “A” contains the most profitable values, i.e., the largest data values (“the important few”), subset “B” contains data for which the profit gain is equal to the effort required to achieve that gain, and subset “C” contains the nonprofitable values, i.e., the lowest probabilities (“the trivial many”).

The threshold ε for a class assignment probability that is considered too low for the assignment decision is defined as the BC limit calculated in the ABC analysis. In this way, the “trivial many” probabilities are removed from the Bayesian class assignment decision. The computed ABC analysis determines the limits for the subsets, in particular for the B–C limit used here, based on results from the statistics for asymmetrical (skewed) distributions, supplemented by the efficiency theory [47]. The theorem of Bayes is not limited to univariate data. Multivariate applications, even in its “naive” form that assumes independence, often show that it can compete with modern AI methods such as support vector machines or modern decision tree algorithms [48]. In the present paper, we also demonstrate the proposed approach for two-dimensional data (Figure 4). However, the method can be applied unchanged to multivariate classification tasks.

#### 4.2.2. Corrected Assignments to Classes

Cases that have a probability of belonging to a particular class below the threshold ε calculated above can either be left “unclassified”, which is called “reasonable Bayes” classification, or their classification can be based on the distance to the class center of neighborhood classified cases, which is called “plausible Bayes” classification.

#### Reasonable Bayes

In order to achieve reasonable assignments to the classes with Bayes’ theorem, i.e., the evidence, the sum of the class likelihoods weighted with the priors is considered. If the evidence falls below a threshold ε, the conclusion, i.e., class assignment, becomes uncertain. Thus, a Bayes decision is accepted as reasonable, only if there is sufficient evidence for a data value. If the evidence falls below ε, the decision is suspended, i.e., a special value, such as SQL’s “NULL” [49], or the symbol “NaN” (not a number) proposed by IEEE for missing values in computations (IEEE Standard for Floating-Point Arithmetic, 2019), is the result of the decision. Suggested estimates for ε are, first, the BC limit calculated during the ABC analysis as the maximum value in set C that captures the so-called “trivial many” [46], and second, ε = 1% of the maximum evidence. For normal distributions, the computed ABC analysis captures approximately the range m ± 2 s and the 1% limit the range m ± 3 s [50].

#### Plausible Bayes

Often a decision is required for all empirical cases, even for cases x with low evidence. A reasonable assignment is then to assign x to the class whose probability centroid is closest. In general, this can be calculated using Voronoi cells [45] that are defined as follows. Let *P* = {$p_{i},\;i=1,\cdots ,n$} be a set of *n* distinct points in a metric space D ϵ$R^{d}$ with a distance function d(x,y) defined for all *x,y* in *P*. The Voronoi cells of *P* are a tessellation of D into *n* cells, one for each point in P. A point *x* lies in the cell corresponding to a (center) point $p_{i}\;\epsilon \;P$, if for each $p_{i}\;\epsilon \;P,\;j\neq i:\;d\left(x,p_{j}\right)>\;d\left(x,p_{i}\right)$. The center points for Voronoi cells of distributions are the expected value $E\left[X\right]=\int_{-\infty }^{\infty }xf\left(x\right)dx$. For normal distributions, this is the mean *m*.

In the one-dimensional case, a “plausible Bayes” classification is computed using the distances from x to the modes (=maxima) or mean values {m${}_{1}$, ⋯, m${}_{c}$} of the pdfs of the class likelihoods. If the “plausible Bayes” calculation results in an undefined value, the class with the closest distance is assigned to x: class(x) = argmin({d(x,m${}_{1}$), …, d(x,m${}_{c}$)}). There are efficient algorithms for Voronoi cell calculations for feature spaces D ϵ$R^{d}$ with d from 1 up to 20, which are included in most statistical software packages, for example in the R package “geometry” (https://CRAN.R-project.org/package=geometry (accessed on 18 September 2022) [51]) or as built-in function of the Matlab system. Many of these algorithms are based on the efficient and well-tested convex hull algorithm “qhull” (http://www.qhull.org (accessed on 18 September 2022) [52]). For larger number of features (d > 20), there are approximations for the Voronoi cells or their equivalent, the Delaunay graph (e.g., [53]).

### 4.3. Experimentation

Programming was performed in the matrix laboratory programming language using the Matlab software package (version 7.1, MathWorks for Windows, Natick, MS, USA), in the R language [54] using the R software package [12], version 4.1.3 for Linux, available free of charge from the Comprehensive R Archive Network (CRAN) at https://CRAN.R-project.org/ (accessed on 18 September 2022), and in the Python language [55] using Python version 3.8.12 for Linux, available free of charge at https://www.python.org (accessed on 18 September 2022). Data sets comprised body heights of school-aged children and adolescents from 1985 to 2019 in 200 countries with 65 million participants [9], which served as introductory example. Further data sets included lipid markers measured in blood serum of patients with multiple sclerosis or healthy controls published previously [14], and public domain flow cytometric data of peripheral blood [19].

## 5. Conclusions

In some real-world cases, Bayesian reasoning leads to implausible results. This effect occurs particularly with data with a small general probability (evidence). Using item categorization via a computed ABC analysis on the evidence of the data, the proposed method at least allowed to identify which data might be misclassified and for which data Bayes’ theorem provided good classifications. A threshold ε was calculated for the probability of class assignment to be considered as too low to classify. The “reasonable Bayes” classification proposed to leave those points unclassified or classified as “uncertain”. The “plausible Bayes” classification proposed that data points that were not assigned to a class using the “plausible Bayes” method be placed in the Voronoi cell of the class centers. Thus, the proposed extension of a Bayesian inference of class membership can be used to obtain robust and plausible class assignments even for data at the extremes of the distribution and/or for which evidence is weak. The gain in assignment accuracy depends on the actual data set and ranged in the present experiments from as little as 1% or less to as much as 10%.

## Data Availability

The data used in the experiments in this report are publicly available and referenced accordingly.

## References

[1] Bayes M., Price M. (1763). An Essay towards Solving a Problem in the Doctrine of Chances. By the Late Rev. Mr. Bayes, F. R. S. Communicated by Mr. Price, in a Letter to John Canton, A. M. F. R. S. Philos. Trans..

[2] Tiberi S., Walsh M., Cavallaro M., Hebenstreit D., Finkenstädt B. (2018). Bayesian inference on stochastic gene transcription from flow cytometry data. Bioinformatics.

[3] Yu J.S., Pertusi D.A., Adeniran A.V., Tyo K.E.J. (2017). CellSort: A support vector machine tool for optimizing fluorescence-activated cell sorting and reducing experimental effort. Bioinformatics.

[4] Džunková M., Moya A., Vázquez-Castellanos J.F., Artacho A., Chen X., Kelly C., D’Auria G. (2016). Active and Secretory IgA-Coated Bacterial Fractions Elucidate Dysbiosis in Clostridium difficile Infection. mSphere.

[5] Comella P.H., Gonzalez-Kozlova E., Kosoy R., Charney A.W., Peradejordi I.F., Chandrasekar S., Tyler S.R., Wang W., Losic B., Zhu J. (2021). A Molecular network approach reveals shared cellular and molecular signatures between chronic fatigue syndrome and other fatiguing illnesses. medRxiv.

[6] Kovalchik S. RISmed: Download Content from NCBI Databases, 2020. https://CRAN.R-project.org/package=RISmed.

[7] Perfors A., Tenenbaum J.B., Griffiths T.L., Xu F. (2011). A tutorial introduction to Bayesian models of cognitive development. Cognition.

[8] Gelman A., Yao Y. (2020). Holes in Bayesian statistics. J. Phys. G Nucl. Part. Phys..

[9] Rodriguez-Martinez A., Zhou B., Sophiea M.K., Bentham J., Paciorek C.J., Iurilli M.L.C., Carrillo-Larco R.M. (2020). Height and body-mass index trajectories of school-aged children and adolescents from 1985 to 2019 in 200 countries and territories: A pooled analysis of 2181 population-based studies with 65 million participants. Lancet.

[10] Wang Y., Ma Y., Carroll R.J. (2009). Variance estimation in the analysis of microarray data. J. R. Stat. Soc. Ser. B Stat. Methodol..

[11] Archambeau C., Verleysen M. (2007). Robust Bayesian clustering. Neural Netw..

[12] R Core Team (2008). R: A Language and Environment for Statistical Computing.

[13] Wickham H. (2009). ggplot2: Elegant Graphics for Data Analysis.

[14] Lotsch J., Schiffmann S., Schmitz K., Brunkhorst R., Lerch F., Ferreiros N., Wicker S., Tegeder I., Geisslinger G., Ultsch A. (2018). Machine-learning based lipid mediator serum concentration patterns allow identification of multiple sclerosis patients with high accuracy. Sci. Rep..

[15] Sisignano M., Angioni C., Ferreiros N., Schuh C.D., Suo J., Schreiber Y., Dawes J.M., Antunes-Martins A., Bennett D.L., McMahon S.B. (2013). Synthesis of lipid mediators during UVB-induced inflammatory hyperalgesia in rats and mice. PLoS ONE.

[16] Zschiebsch K., Fischer C., Pickert G., Haeussler A., Radeke H., Grosch S., Ferreiros N., Geisslinger G., Werner E.R., Tegeder I. (2016). Tetrahydrobiopterin attenuates DSS-evoked colitis in mice by rebalancing redox and lipid signaling. J. Crohns. Colitis..

[17] Blachnio-Zabielska A.U., Chacinska M., Vendelbo M.H., Zabielski P. (2016). The Crucial Role of C18-Cer in Fat-Induced Skeletal Muscle Insulin Resistance. Cell. Physiol. Biochem..

[18] Rossi C., Cicalini I., Zucchelli M., di Ioia M., Onofrj M., Federici L., Del Boccio P., Pieragostino D. (2018). Metabolomic Signature in Sera of Multiple Sclerosis Patients during Pregnancy. Int. J. Mol. Sci..

[19] Thrun M., Hoffmann J., Rohnert M., von Bonin M., Oelschlägel U., Brendel C., Ultsch A. (2022). Flow Cytometry datasets consisting of peripheral blood and bone marrow samples for the evaluation of explainable artificial intelligence methods. Mendeley Data.

[20] Frearson J.A., Alexander D.R. (1996). Protein tyrosine phosphatases in T-cell development, apoptosis and signalling. Immunol. Today.

[21] Woodhead V.E., Stonehouse T.J., Binks M.H., Speidel K., Fox D.A., Gaya A., Hardie D., Henniker A.J., Horejsi V., Sagawa K. (2000). Novel molecular mechanisms of dendritic cell-induced T cell activation. Int. Immunol..

[22] Horikoshi A., Sawada S., Endo M., Kawamura M., Murakami J., Iizuka Y., Takeuchi J., Ohshima T., Horie T., Motoyoshi K. (1995). Relationship between responsiveness to colony stimulating factors (CSFs) and surface phenotype of leukemic blasts. Leuk. Res..

[23] Rosenzwajg M., Tailleux L., Gluckman J.C. (2000). CD13/N-aminopeptidase is involved in the development of dendritic cells and macrophages from cord blood CD34+ cells. Blood.

[24] Herzenberg L.A., Tung J., Moore W.A., Herzenberg L.A., Parks D.R. (2006). Interpreting flow cytometry data: A guide for the perplexed. Nat. Immunol..

[25] Verschoor C.P., Lelic A., Bramson J.L., Bowdish D.M. (2015). An Introduction to Automated Flow Cytometry Gating Tools and Their Implementation. Front. Immunol..

[26] Moon T.K. (1996). The expectation-maximization algorithm. IEEE Signal Process. Mag..

[27] Waskom M.L. (2021). seaborn: Statistical data visualization. J. Open Source Softw..

[28] Akaike H. (1974). A new look at the statistical model identification. IEEE Trans. Aut. Control.

[29] Pedregosa F., Varoquaux G., Gramfort A., Michel V., Thirion B., Grisel O., Blondel M., Prettenhofer P., Weiss R., Dubourg V. (2011). Scikit-learn: Machine Learning in Python. J. Mach. Learn. Res..

[30] Devroye L., Gyorfi L., Lugosi G. (1996). A Probabilistic Theory of Pattern Recognition.

[31] Hastie T., Tibshirani R., Friedman J.H. (2009). The Elements of Statistical Learning: Data Mining, Inference, and Prediction.

[32] Piryonesi S.M., El-Diraby Tamer E. (2020). Role of Data Analytics in Infrastructure Asset Management: Overcoming Data Size and Quality Problems. J. Transp. Eng. Part B Pavements.

[33] Ziemski M., Wisanwanichthan T., Bokulich N.A., Kaehler B.D. (2021). Beating Naive Bayes at Taxonomic Classification of 16S rRNA Gene Sequences. Front. Microbiol..

[34] Ontivero-Ortega M., Lage-Castellanos A., Valente G., Goebel R., Valdes-Sosa M. (2017). Fast Gaussian Naïve Bayes for searchlight classification analysis. Neuroimage.

[35] Griffis J.C., Allendorfer J.B., Szaflarski J.P. (2016). Voxel-based Gaussian naïve Bayes classification of ischemic stroke lesions in individual T1-weighted MRI scans. J. Neurosci. Methods.

[36] Zhang H., Cao Z.X., Li M., Li Y.Z., Peng C. (2016). Novel naïve Bayes classification models for predicting the carcinogenicity of chemicals. Food Chem. Toxicol..

[37] Ahmed M.S., Shahjaman M., Rana M.M., Mollah M.N.H. (2017). Robustification of Naïve Bayes Classifier and Its Application for Microarray Gene Expression Data Analysis. BioMed Res. Int..

[38] Zimek A., Schubert E., Kriegel H.P. (2012). A survey on unsupervised outlier detection in high-dimensional numerical data. Stat. Anal. Data Min. ASA Data Sci. J..

[39] Mor B., Garhwal S., Kumar A. (2020). A Systematic Review of Hidden Markov Models and Their Applications. Arch. Comput. Methods Eng..

[40] Freedman D. (2012). Markov Chains.

[41] Li Q., Li R., Ji K., Dai W. Kalman Filter and Its Application. Proceedings of the 2015 8th International Conference on Intelligent Networks and Intelligent Systems (ICINIS).

[42] Fischer H. (2011). A History of the Central Limit Theorem: From Classical to Modern Probability Theory.

[43] Minker J., Loveland D.W. (1982). On indefinite databases and the closed world assumption. 6th Conference on Automated Deduction. CADE 1982.

[44] Ultsch A., Thrun M.C., Hansen-Goos O., Lotsch J. (2015). Identification of Molecular Fingerprints in Human Heat Pain Thresholds by Use of an Interactive Mixture Model R Toolbox (AdaptGauss). Int. J. Mol. Sci..

[45] Voronoi G. (1908). Nouvelles applications des paramètres continus à la théorie des formes quadratiques. Premier mémoire. Sur quelques propriétés des formes quadratiques positives parfaites. J. FüR Die Reine Und Angew. Math. (Crelles J.).

[46] Ultsch A., Lotsch J. (2015). Computed ABC Analysis for Rational Selection of Most Informative Variables in Multivariate Data. PLoS ONE.

[47] Wood J.C., Wood M.C. (2005). Joseph M. Juran: Critical Evaluations in Business and Management.

[48] Zhang H. The Optimality of Naive Bayes. Proceedings of the Seventeenth International Florida Artificial Intelligence Research Society Conference.

[49] van der Meyden R., Chomicki J., Saake G. (1998). Logical Approaches to Incomplete Information: A Survey. Logics for Databases and Information Systems.

[50] Reosekar R.S., Pohekar S.D. (2014). Six Sigma methodology: A structured review. Int. J. Lean Six Sigma.

[51] Habel K., Grasman R., Gramacy R.B., Mozharovskyi P., Sterratt D.C. Geometry: Mesh Generation and Surface Tessellation, 2019. https://CRAN.R-project.org/package=geometry.

[52] Barber C.B., Dobkin D.P., Huhdanpaa H. (1996). The quickhull algorithm for convex hulls. ACM Trans. Math. Softw..

[53] Polianskii V., Pokorny F.T. (2020). Voronoi Graph Traversal in High Dimensions with Applications to Topological Data Analysis and Piecewise Linear Interpolation. Proceedings of the 26th ACM SIGKDD International Conference on Knowledge Discovery &Data Mining.

[54] Ihaka R., Gentleman R. (1996). R: A Language for Data Analysis and Graphics. J. Comput. Graph. Stat..

[55] Van Rossum G., Drake F.L. (1995). Python Tutorial.

